# IMP3 Protein Overexpression Is Linked to Unfavorable Outcome in Laryngeal Squamous Cell Carcinoma

**DOI:** 10.3390/cancers13174306

**Published:** 2021-08-26

**Authors:** Diana Maržić, Blažen Marijić, Tamara Braut, Stefan Janik, Manuela Avirović, Ita Hadžisejdić, Filip Tudor, Katarina Radobuljac, Miran Čoklo, Boban M. Erovic

**Affiliations:** 1 Department of Audiology and Phoniatrics, Clinical Hospital Center Rijeka, 51000 Rijeka, Croatia; diana.marzic@uniri.hr (D.M.); audiologija-tajnistvo@kbc-rijeka.hr (K.R.); 2 Faculty of Medicine, University of Rijeka, 51000 Rijeka, Croatia; blazen.marijic@uniri.hr (B.M.); tamara.braut@uniri.hr (T.B.); manuela.avirovic@medri.uniri.hr (M.A.); ita.hadzisejdic@medri.uniri.hr (I.H.); filip.tudor@medri.uniri.hr (F.T.); 3 Institute of Head and Neck Diseases, Evangelical Hospital, 1180 Vienna, Austria; 4 Department of Otorhinolaryngology, Head and Neck Surgery, Clinical Hospital Center Rijeka, 51000 Rijeka, Croatia; 5 Department of Otorhinolaryngology, Head and Neck Surgery, Medical University Vienna, 1190 Vienna, Austria; stefan.janik@meduniwien.ac.at; 6 Clinical Department of Pathology and Cytology, Clinical Hospital Center Rijeka, 51000 Rijeka, Croatia; 7 Center for Applied Bioanthropology, Institute for Anthropological Research, 10000 Zagreb, Croatia; miran.coklo@inantro.hr

**Keywords:** IMP3, laryngeal carcinoma, dysplasia, immunohistochemistry, survival

## Abstract

**Simple Summary:**

IMP3 expression was analyzed in patients with malignant (laryngeal squamous cell carcinoma), semi-malignant (dysplasia) and benign (nodules, polyps) laryngeal lesions and correlated with clinical characteristics. Higher IMP3 stains were particularly found in malignant laryngeal pathologies, which might be useful for differentiation between premalignant and malignant lesions. In laryngeal cancer patients, higher IMP3 expression was associated with positive neck nodes and worse disease-specific survival.

**Abstract:**

Background: The aim of this study was to (i) determine IMP3 protein expression in benign and malignant laryngeal lesions, (ii) compare its expression to Ki-67, p53, cyclin D1, and (iii) finally, to examine the prognostic power of IMP3 in squamous cell carcinomas of the larynx (LSSC). Methods: IMP3 protein expression was evaluated in 145 patients, including 62 LSCC, 45 dysplasia (25 with low and 20 with high-grade dysplasia), and 38 benign lesions (vocal cord polyps and nodules). Results: IMP3 was significantly higher expressed in LSCC compared to dysplasia and benign lesions (*p* < 0.001; *p* < 0.001, respectively). Similarly, higher expression patterns were observed for Ki-67 and p53, whereas cyclin D1 was equally distributed in all three lesions. IMP3 (*p* = 0.04) and Ki-67 (*p* = 0.02) expressions were significantly linked to neck node positivity, and IMP3 overexpression to worse disease-specific survival (*p* = 0.027). Conclusion: Since IMP3 showed significantly higher expression in laryngeal carcinomas, but not in high- or low-grade dysplasia, it serves as a useful marker to differentiate between invasive and noninvasive lesions. Higher IMP3 expression represented a significantly worse prognosticator for clinical outcomes of patients with squamous cell carcinoma of the larynx.

## 1. Introduction

Laryngeal squamous cell carcinoma (LSCC) remains a highly morbid and fatal disease whose overall survival of 60% has been constantly high for more than half a century [1,2,3,4,5]. To improve clinical outcomes, new therapeutical and diagnostic options are strongly needed [6,7]. A better understanding of tumor cell biology is the main driver leading to the discovery of a new generation of drugs that act at the molecular level to prevent the growth and spread of tumors [8]. Although these are newer drugs, some of them have already found their place in the therapeutic protocols in a variety of human cancers, including LSCC [9,10]. Important progress in the treatment of head and neck squamous cell carcinoma (HNSCC) has been made with the discovery of anti-EGFR and anti-PD-L1 therapy and their implementation into treatment protocols [9,10,11]. Although the survival of a certain group of patients was thus improved, the overall oncological outcome is not satisfactory, especially in advanced stages or recurrent and/or metastatic disease [10]. In addition to the importance of biomarkers as targeted molecules in anti-tumor therapy, they also play a very important role in the early diagnosis of disease, monitoring the effect of treatment, prognosis, and prevention [11].

One of the novel biomarkers possibly involved in the carcinogenesis of many tumors, including HNSCC, is the insulin-like growth factor II m-RNA-binding protein 3 (IMP3) [12]. As a member of the IMP family, it is linked to cell migration, embryogenesis, and tumorigenesis [12,13]. IMP3 overexpression occurs in various important cancer types including HNSCC and is linked to aggressive tumor features [14]. Data in the literature on the role of this marker in laryngeal carcinogenesis are scarce, and studies that systematically show the impact of this marker on the outcome of the disease are particularly absent lacking. Furthermore, data on the expression of IMP3 in precancerous or benign lesions vary. While some authors state that this is a marker that distinguishes malignant from benign lesions [15], others also prove its presence in premalignant and nonmalignant lesions [16,17,18,19].

Precisely because of the above-mentioned facts, the goals of our study were (i) to determine the expression of IMP3 in benign laryngeal lesions, low and high-grade dysplasia, and LSCC; (ii) correlate its expression with other markers of cell growth and proliferation (Ki-67, p53 and cyclin D1); (iii) determine pathological–clinical features of the tumor with IMP3 overexpression and (iv) its influence on disease outcome of patients with laryngeal squamous cell carcinoma.

## 2. Materials and Methods

### 2.1. Study Cohort

This retrospective study was conducted at the Department of Otorhinolaryngology and Head and Neck Surgery, Clinical Hospital Center Rijeka, and at the Department of General Pathology and Pathological Anatomy, Faculty of Medicine, the University of Rijeka from 2010 till 2021. Initially, our cohort consisted of the same 153 patients who have been previously analyzed [20]. After applying more stringent inclusion criteria, 3 patients were excluded for subsequent analysis, as well as 9 additional samples because of faded stains without reliable expression patterns. Conversely, 4 patients with dysplasia could meanwhile be included. Finally, a total of 145 tissue samples with associated clinical and pathological data were evaluated, out of which 38 were benign lesions (vocal polyps and nodules), 45 dysplasia (25 low and 20 high grade), and 62 LSCC. Exclusion criteria included prior treatment with radiochemotherapy, immunotherapy, surgery for head and neck malignancy or a previous tumor of other sites. Clinical and pathohistological data relevant to the study, which were collected from medical records, were evaluated.

### 2.2. Immunohistochemistry

Tissue microarrays (TMAs) were constructed using triplicates of 1 mm cores of the abovementioned samples. The following antibodies were used in the study: (i) for Ki-67 (MIB-1, 1:100; DakoCytomation, Glostrup, Denmark); (ii) for IMP3 (Clone 69.1, 1:100; Dako Cytomation, Glostrup, Denmark); (iii) for p53 (DO-7, ready to use, Dako Cytomation, Glostrup, Denmark); (iv) for cyclin D1 (Ep 12, 1:50; Dako Cytomation, Glostrup, Denmark). Antigen retrieval protocol, incubation, and other steps of immunohistochemistry (IHC) method for sample preparation were performed following the manufacturer’s instructions and are described in detail in our previous studies [20,21,22,23,24,25,26].

### 2.3. Evaluation of Immunoreactivity

Slides were reviewed independently by two pathologists I.H. and M.A. We used a qualitative score to evaluate the staining intensity of p53, cyclin D1 and IMP3 in tissue specimens. Staining intensity was quantified as none (0), weak (1); faintly staining visible at low magnification of light microscope), moderate (2); moderate staining visible at low magnification of light microscope), and strong (3); strong staining visible at low magnification of light microscope), and multiplied by the number of positively stained cells (range 0–100%), resulting in scores ranging from 0 to 300. Regarding Ki-67, we only counted the number of positively stained cells. The median p53 (55.05), cyclin D1 (111.45), and Ki-67 (25.0) scores were used for dichotomizing patients into a high and a low cohort. We plotted the receiver operating characteristic (ROC) curve and calculated the Youden index to identify an optimal IMP3 cut-off value of 192.2 to differentiate between high and low IMP3 subgroups. The AUC value was calculated at 0.617, while Youden index was 0.257.

### 2.4. Statistical Analysis

Statistical analyses were performed using SPSS version 27.0 software (IBM SPSS Inc., Armonk, NY, USA). Unless otherwise specified, data are reported as mean ± standard deviation (SD). The Chi-square test was used to investigate the association between nominal variables. Unpaired Student’s *t*-test and one-way ANOVA were used to compare means of two or more two-independent groups with normal (Gaussian) distributions. Post hoc comparisons were computed with Tukey’s B and Bonferroni correction. Pearson correlation (r) was performed to analyze relationships between two numerical variables. We plotted the receiver operating characteristic (ROC) curve and calculated the Youden-index to identify an optimal IMP3 cut-off value of 192.2 to dichotomize patients into a high and low expressing subgroup. Kaplan–Meier analyses and log-rank tests were assessed for univariate outcome analysis. Uni- and multivariate cox regression analyses were used to evaluate the prognostic impact of different clinical variables on disease-free survival and disease-specific survival. Hazard ratios (HRs) and corresponding 95% confidence intervals (CIs) are indicated. All tests were performed two-sided, and *p*-values below 0.05 were considered statistically significant.

## 3. Results

### 3.1. Study Cohort

Our study included 123 male and 22 female patients with a mean age of 57 ± 13.4 years. The group of dysplasia was stratified according to the WHO classification into low- (*n* = 25) and high-grade (*n* = 20) lesions. In the SCC group, 10 (16.1%) T1, 11 (17.7%) T2, 28 (45.2%) T3, and 13 (21%) T4 tumors were diagnosed with positive neck nodes in 12 (19.4%) patients. Subsequently, 22 (35.5%) patients were classified to be in early (I and II) and 40 (65.5%) patients in advanced-stage disease (III and IV). Postoperative radiotherapy was applied in 33 (54.1%) patients. Additional demographic characteristics of the LSCC group are shown in Table 1.

### 3.2. IMP3, Ki-67, p53, and Cyclin D1 Expression

**Figure 1:**
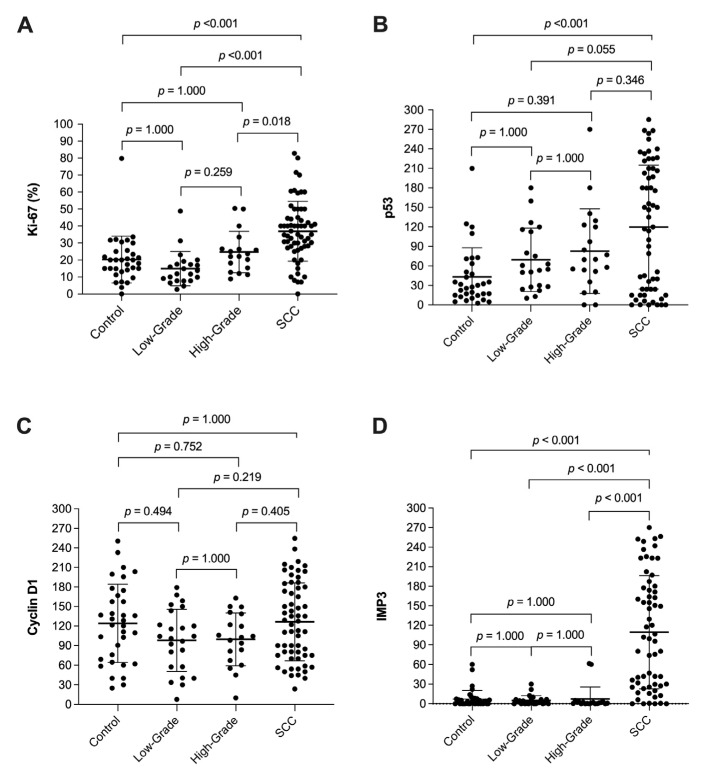
Box plots for post hoc comparison: Plots are indicated as mean ± SD. They show the expression of Ki-67 (**A**), p53 (**B**), cyclin D1 (**C**), and IMP3 (**D**) proteins in laryngeal lesions.

**Figure 2:**
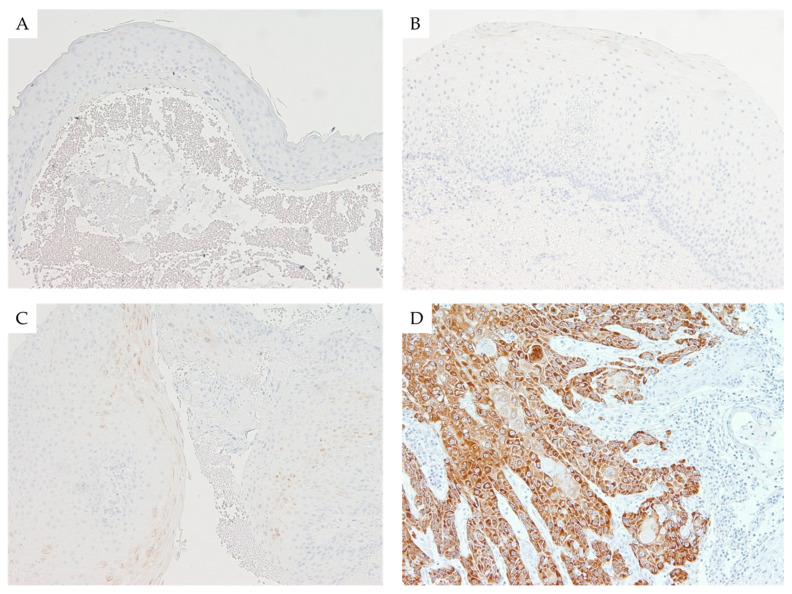
Immunohistochemical expression of IMP3 in laryngeal lesions: Comparison of focal and weak immunohistochemical cytoplasmic staining against IMP3 in laryngeal nodule (**A**), low-grade dysplasia (**B**), and high-grade dysplasia (**C**) versus diffuse and strong cytoplasmic staining in laryngeal squamous cell carcinoma (LSCC) (**D**); (100× magnification).

The mean percentage of Ki-67 positive cells was significantly different between the LSCC group and high and low-grade dysplasia, as well as when comparing to benign lesions (*p* < 0.001), while no significant difference was found among the subgroups of dysplasia or when comparing them to benign lesions (*p* = 0.259, *p* = 1.000, *p* = 1.000, retrospectively). Using other markers, a comparison of mean scores (range 0 to 300) was evaluated, and a statistically significant difference was observed between LSCC versus dysplasia and the control group in p53 (*p* < 0.001) and IMP3 (*p* < 0.001) after immunohistochemistry analysis. Importantly, IMP3 expression was significantly higher solely in invasive (LSCC) but almost unchanged in noninvasive cases, including benign lesions and patients with dysplasia as well. Regarding cyclin D1 mean scores, we did not find any statistically significant differences between the observed groups (*p* = 0.079). The results of these analyses are shown in Table 2. Post hoc comparisons are illustrated in Figure 1 and patterns of immunohistochemical staining are shown in Figure 2.

### 3.3. Correlation of IMP3 with Ki-67, p-53, Cyclin D1, and Histopathological and Clinical Data

According to the Pearson correlation analysis, IMP3 expression significantly correlated with other proliferation markers, such as Ki-67, p53, and cyclin D1 (*p* < 0.001, *p* < 0.001, *p* = 0.026; respectively) (Table 3). Additionally, a statistically significant positive correlation was observed for Ki-67 versus p53 and cyclin D1 (*p* < 0.001, *p* = 0.007, respectively), as well as between p53 and cyclin D1 (*p* < 0.001).

Comparing the clinical and pathological features of LSCC (Table 4), we found an overexpression of IMP3 and Ki-67 in those tumors that metastasized to regional neck lymph nodes (*n* = 12 or 19.35%; *p* = 0.04; *p* = 0.02, respectively).

### 3.4. Impact of IMP3 on Disease-Free Survival (DFS) and Disease-Specific Survival (DSS)

**Figure 3:**
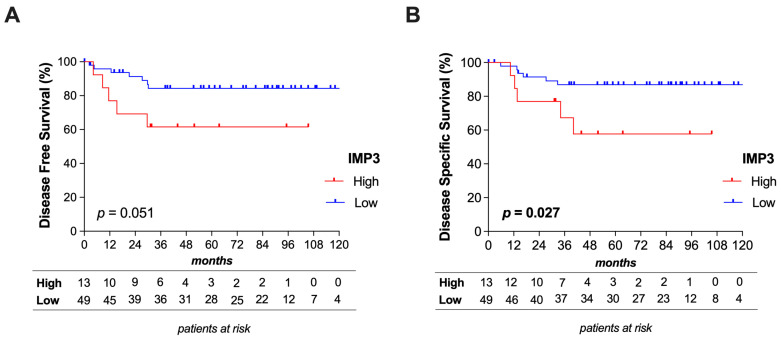
Survival curves: Kaplan–Meier plots illustrating a trend toward shorter disease-free survival in patients with high IMP3 expression (**A**) and a significantly worse disease-specific survival (DSS) (**B**).

Kaplan–Meier plots illustrate a trend that patients with a high IMP3 expression carried a shorter time to recurrence (*p* = 0.051) and a significantly worse disease-specific survival (*p* = 0.027) (Figure 3). The predictive power of different clinical variables was evaluated regarding disease-free survival (DFS) and disease-specific survival (DSS) (Table 5). According to univariate analysis, disease progression to regional lymph nodes (*p* = 0.014; HR = 4.44), poor tumor differentiation (*p* = 0.046; HR = 2.541), and tumors with higher IMP3 expression (*p* = 0.038; HR = 3.509) had a significant impact on patients’ DSS. Furthermore, disease progression to regional lymph nodes (*p* = 0.022; HR = 3.817) had also an impact on DFS. After multivariate analysis, none of the factors proved to be an independent predictive marker for DFS or DSS.

## 4. Discussion

IMP3 is a small oncofetal protein composed of 580 amino acids and encoded by the *IMP3 gene* located on chromosome 7 [27]. Although it represents a relatively novel biomarker discovered in the late 1990s, it is known to participate in human embryogenesis and carcinogenesis [28,29]. Its important role in early embryogenesis is manifested through participation in the processes of RNA trafficking and stabilization, cell growth, and migration [12,13,27,30]. In that period, the expression of IMP3 can be detected in the fetal tissue of the placenta, muscles, epithelial cells [27,29], while in adulthood, IMP3 can be found in the placenta, gonads, tonsillar tissue, and lymph nodes [29]. Re-expression occurs again with the appearance of malignant cells, which has been reported in a large variety of human cancers [12].

In terms of its clinical application, the question arises in which moment of carcinogenesis the expression of IMP3 begins to manifest itself in laryngeal lesions, and when it could be a predictor of malignancy. Therefore, in this study, we included groups of low- and high-grade dysplasia, as well as a control group consisting of benign lesions such as laryngeal polyps. The low expression of IMP3 was observed in benign lesions and dysplasia, while diffuse and strong cytoplasmic staining was found in LSCCs. A similar observation was made in our previous study [20]. Subsequently, we suggest that IMP3 might be useful in differentiating between nonmalignant and malignant laryngeal lesions in the future. Our findings are in agreement with the results of Chen et al. obtained on 238 laryngeal lesions, out of which 227 were LSCC and 11 were dysplasia [15]. They demonstrated that IMP3 is a highly sensitive and specific biomarker for LSCC and that evaluating its expression can help pathologists to distinguish benign and precancerous lesions from malignant entities, which is especially useful when analyzing small biopsies [15]. Moreover, IMP3 expression can serve as an auxiliary tool to pave the path for a safer and more reliable final diagnosis. Although our study supports Chen’s results, more studies are needed to underline this observation on LSCC [15], as studies on dysplasia [16,17,18] and some benign lesions [19] of other locations found increased IMP3 expression.

Furthermore, we analyzed the association of IMP3 protein expression with the clinical and pathological features of the tumor. We proved that tumors that spread to regional lymph nodes of the neck had a statistically significant higher expression of IMP3 protein, compared to those in which the tumor was restricted to the larynx only. Evidence from the literature indicates that IMP3 promotes cell migration and invasion, which requires cooperation with a whole range of agents at the molecular level [12,29]. Our results further support a study by Jiang et al. from 2006, in which patients with renal cancer metastases had a stronger expression of IMP3 protein, compared to those with localized disease [31]. Furthermore, Jiang et al. pointed out that IMP3 may serve as an independent prognostic factor in predicting metastatic disease [31]. The potential benefit for LSCC patients could also be in the treatment of the N0 neck, for which, with the help of IMP3 expression, we can predict the occurrence of regional metastases that require selective neck dissection. Again, further studies on a larger series of patients are needed to validate the prognostic and diagnostic value of IMP3 in this context.

When comparing IMP3 with markers of cell proliferation and cell cycle, in particular Ki-67, p53, and cyclin D1, we obtained a positive statistically significant correlation, which indicates that IMP3 synchronously acts with these three markers promoting uncontrolled cell growth and proliferation, as well as inhibition of apoptosis [29]. A study by Er et al. demonstrated that inhibition of IMP3 expression leads to downregulation of proteins associated with cell proliferation such as Ki-67 and epidermal growth factor receptor (EGFR) and thus imposes the possible importance of the IMP3 molecule as a promising therapeutic target [32]. Further studies are needed in this regard as well, to precisely clarify the molecular mechanism behind its impact on cancer progression.

In the recent meta-analysis by Chen et al. with 53 included studies containing 8937 patients, the association between high IMP3 expression and effects on survival in solid tumors of different sites was analyzed proving that strong expression of IMP3 was associated with poor disease-free and recurrence-free survival [12]. However, there was a lack of data to confirm this for LSCC. Therefore, our study is, to the best of our knowledge, the first analyzing the impact of IMP3 expression on disease-free survival in LSCC patients. Given that the result is at the trend level (*p* = 0.051) and that we proved it by univariate but not multivariate analysis, it is difficult to draw a clear conclusion that IMP3 is an indicator of poorer survival; therefore, further research is necessary.

## 5. Conclusions

We demonstrated, for the first time, the possible role of IMP3 overexpression on disease-free survival in LSCC patients and its elevated expression in cases with lymph node metastases. We also confirmed previous findings on the usefulness of IMP3 in distinguishing malignant versus premalignant and nonmalignant lesions. Additional studies are certainly needed to implement IMP3 into protocols for diagnosis and follow-up of LSCC patients.

## Figures and Tables

**Table 1:** Additional demographic characteristics of the LSCC group.

Variable		Supraglottic Tumors	GlotticTumors	Subglottic Tumors	Transglottic Tumors	*p*
Sex	Female	0	2	0	3	0.900
	Male	1	30	1	25	
Age	<59.3 year	1	11	0	5	0.165
	≥59.3 year	0	21	1	23	
Smoking	Yes	1	26	0	23	0.216
	No	0	6	1	5	
Alcohol	Yes	1	16	0	16	0.508
	No	0	16	1	12	
Smoking and alcohol	Yes	1	14	0	15	0.461
	No	0	18	1	13	

**Table 2:** Comparison of mean values of Ki-67, p53, cyclin D1, and IMP3 protein expression in laryngeal lesions.

Variable	Control Group	Low-Grade Dysplasia	High-Grade Dysplasia	SCC	*p*
Ki-67	20.3 ± 13.74	14.9 ± 10.06	24.7 ± 12.16	36.9 ± 17.65	*p* < 0.001
P53	43.0 ± 43.0	69.4 ± 48.58	82.7 ± 65.22	119.8 ± 95.27	*p* < 0.001
Cyclin D1	124.0 ± 60.1	98.1 ± 47.62	99.5 ± 40.46	126.5 ± 59.73	*p* = 0.079
IMP3	7.29 ± 13.14	4.95 ± 7.31	7.25 ± 18.32	109.5 ± 86.60	*p* < 0.001

**Table 3:** Pearson correlations between examined markers.

Variable		Ki-67	p53	Cyclin D1	IMP3
Ki-67	Pearson correlation	1	0.429 **	0.232 **	0.497 **
	Sig. (two- tailed)		0.000	0.007	0.000
	*n*	134	126	132	134
p53	Pearson correlation	0.429 **	1	0.320 **	0.433 **
	Sig. (two- tailed)	0.000		0.000	0.000
	*n*	126	132	130	132
Cyclin D1	Pearson correlation	0.232 **	0.320 **	1	0.190 *
	Sig. (two- tailed)	0.007	0.000		0.026
	*n*	132	130	137	137
IMP3	Pearson correlation	0.497 **	0.433 **	0.190 *	1
	Sig. (two- tailed)	0.000	0.000	0.026	
	*n*	134	132	137	145

* Correlation is significant at the 0.05 level (2-tailed). ** Correlation is significant at the 0.01 level (2-tailed).

**Table 4:** Correlation of Ki-67, p53, cyclin D1, and IMP3 markers with clinicopathologic features of the examined group.

			Patients	Ki-67		p53		Cyclin D1		IMP3	
Variable			Number	Mean ± SD	*p*	Mean ± SD	*p*	Mean ± SD	*p*	Mean ± SD	*p*
Sex											
	Male		123	36.5 ± 18.0		118.1 ± 94.0		123.0 ± 59.2		109.0 ± 86.8	
	Female		22	41.8 ± 13.3	0.980	138.1 ± 118.9	0.519	164.3 ± 58.6	0.387	115.4 ± 93.6	0.541
Age											
	≥59.3		72	37.4 ± 17.9		126.7 ± 101.2		133.6 ± 60.4		106.0 ± 87.1	
	<59.3		73	35.5 ± 17.6	0.773	101.1 ± 77.0	0.278	106.7 ± 54.8	0.255	118.7 ± 87.3	0.476
T-Classification											
	T1		10	37.7 ± 22.7		150.8 ± 82.2		112.1 ± 45.2		96.5 ± 71.7	
	T2		11	38.2 ± 8.5		117.7 ± 113.2		145.0 ± 50.8		70.8 ± 60.0	
	T3		28	40.4 ± 20.2		128.7 ± 100.6		133.5 ± 64.3		128.4 ± 96.7	
	T4a		13	27.9 ± 10.3	0.479	81.5 ± 73.0	0.548	106.2 ± 63.7	0.415	111.5 ± 88.9	0.469
N-Classification											
	N0		50	33.5 ± 15.5		121.7 ± 95.9		126.0 ± 55.3		94.3 ± 82.1	
	N+		12	50.8 ± 19.8	0.002	112.4 ± 96.8	0.766	128.3 ± 77.9	0.908	172.6 ± 78.5	0.004
Tumor Stage											
	Stage I		10	37.7 ± 22.7		150.8 ± 82.2		112.1 ± 45.2		96.5 ± 71.7	
	Stage II		12	37.9 ± 8.2		116.1 ± 107.5		148.5 ± 49.9		86.0 ± 77.8	
	Stage III		23	38.9 ± 21.3		133.2 ± 101.6		127.4 ± 59.1		112.6 ± 95.3	
	Stage IV		17	33.1 ± 14.6	0.546	88.5 ± 83.1	0.431	117.3 ± 72.6	0.412	129.6 ± 90.0	0.459
Grading											
	Unknown		4	35.0 ± 30.9		131.2 ± 132.5		118.2 ± 67.5		78.4 ± 65.0	
	G1		12	32.7 ± 19.2		86.2 ± 78.5		131.4 ± 49.0		90.5 ± 84.5	
	G2		33	35.3 ± 12.9		127.8 ± 94.5		116.0 ± 57.9		98.6 ± 76.1	
	G3		13	45.4 ± 21.2	0.345	125.6 ± 103.7	0.753	150.5 ± 69.2	0.456	164.3 ± 104.1	0.382
Recurrence											
	No		50	36.6 ± 17.8		118.0 ± 97.8		125.0 ± 54.5		102.6 ± 85.7	
	Yes		12	38.2 ± 17.8		126.8 ± 88.4		132.2 ± 80.1		138.0 ± 88.1	
		Local	6	33.1 ± 6.5		114.9 ± 111.5		172.0 ± 73.0		157.0 ± 89.1	
		Regional	4	50.5 ± 26.3		140.9 ± 44.3		111.0 ± 83.0		166.9 ± 65.2	
		Distant	2	29.0 ± 15.6	0.156	135.0 ± 127.3	0.170	55.0 ± 1.4	0.410	23.4 ± 33.1	0.468
Survival											
	Alive		33	35.7 ± 15.7		115.8 ± 97.7		134.6 ± 52.9		82.8 ± 76.5	
	Dead		29	38.3 ± 19.8	0.350	123.9 ± 94.3	0.422	117.7 ± 66.1	0.542	139.8 ± 88.6	0.476

**Table 5:** Cox regression analysis.

		**Univariate Analysis**			**Multivariate Analysis**		
**Disease-Specific Survival**		**HR**	***p***	**95% CI**	**HR**	***p***	**95% CI**
	Sex (female vs. male)	0.043	0.484	0.00–294.7			
	Age (<59.3 vs. ≥59.3)	1.563	0.476	0.46–5.35			
	T3-T4a vs. T1-T2	45.45	0.141	0.28–100.0			
	N+ vs. N−	4.44	0.014	1.35–14.7	2.747	0.171	0.65–1.16
	G1 vs. G2 vs. G3 (continuous)	2.541	0.046	1.02–6.35	1.709	0.306	0.61–4.77
	Ki-67 (high vs. low)	0.465	0.223	0.14–1.59			
	p53 (high vs. low)	0.789	0.695	0.24–2.58			
	Cyclin D1 (high vs. low)	0.886	0.841	0.27–2.91			
	IMP-3 (high vs. low)	3.509	0.038	1.07–11.49	1.385	0.692	0.28–6.90
**Disease-Free Survival**							
	Sex (female)	0.043	0.465	0.00–201.2			
	Age (<59.3a)	1.381	0.598	0.42–4.56			
	T3-T4a vs. T1-T2	2.994	0.158	0.65–13.7			
	N+ vs. N−	3.817	0.022	1.21–12.1	3.597	0.173	0.66–10.3
	G1 vs. G2 vs. G3 (continuous)	2.317	0.054	0.99–5.44	1.720	0.270	0.22–4.51
	Ki-67 (high vs. low)	0.389	0.124	0.12–1.30			
	p53 (high vs. low)	0.947	0.927	0.31–2.94			
	Cyclin D1 (high vs. low)	1.046	0.938	0.34–3.25			
	IMP-3 (high vs. low)	2.967	0.064	0.94–9.35	1.205	0.815	0.25–5.68

## Data Availability

The datasets generated and analyzed during the current study are available from the corresponding author on reasonable request.
